# A novel signature predicts prognosis and immunotherapy in lung adenocarcinoma based on cancer-associated fibroblasts

**DOI:** 10.3389/fimmu.2023.1201573

**Published:** 2023-05-31

**Authors:** Qianhe Ren, Pengpeng Zhang, Haoran Lin, Yanlong Feng, Hao Chi, Xiao Zhang, Zhijia Xia, Huabao Cai, Yue Yu

**Affiliations:** ^1^Department of Thoracic Surgery, The First Affiliated Hospital of Nanjing Medical University, Nanjing, China; ^2^Clinical Medical College, Southwest Medical University, Luzhou, China; ^3^Department of General, Visceral, and Transplant Surgery, Ludwig-Maximilians-University, Munich, Germany; ^4^Department of Neurosurgery, First Affiliated Hospital of Anhui Medical University, Hefei, China

**Keywords:** lung adenocarcinoma, fibroblast, prognosis, tumor immune microenvironment, immunotherapy

## Abstract

**Background:**

Extensive research has established the significant correlations between cancer-associated fibroblasts (CAFs) and various stages of cancer development, including initiation, angiogenesis, progression, and resistance to therapy. In this study, we aimed to investigate the characteristics of CAFs in lung adenocarcinoma (LUAD) and develop a risk signature to predict the prognosis of patients with LUAD.

**Methods:**

We obtained single-cell RNA sequencing (scRNA-seq) and bulk RNA-seq data from the public database. The Seurat R package was used to process the scRNA-seq data and identify CAF clusters based on several biomarkers. CAF-related prognostic genes were further identified using univariate Cox regression analysis. To reduce the number of genes, Lasso regression was performed, and a risk signature was established. A novel nomogram that incorporated the risk signature and clinicopathological features was developed to predict the clinical applicability of the model. Additionally, we conducted immune landscape and immunotherapy responsiveness analyses. Finally, we performed *in vitro* experiments to verify the functions of EXO1 in LUAD.

**Results:**

We identified 5 CAF clusters in LUAD using scRNA-seq data, of which 3 clusters were significantly associated with prognosis in LUAD. A total of 492 genes were found to be significantly linked to CAF clusters from 1731 DEGs and were used to construct a risk signature. Moreover, our immune landscape exploration revealed that the risk signature was significantly related to immune scores, and its ability to predict responsiveness to immunotherapy was confirmed. Furthermore, a novel nomogram incorporating the risk signature and clinicopathological features showed excellent clinical applicability. Finally, we verified the functions of EXP1 in LUAD through *in vitro* experiments.

**Conclusions:**

The risk signature has proven to be an excellent predictor of LUAD prognosis, stratifying patients more appropriately and precisely predicting immunotherapy responsiveness. The comprehensive characterization of LUAD based on the CAF signature can predict the response of LUAD to immunotherapy, thus offering fresh perspectives into the management of LUAD patients. Our study ultimately confirms the role of EXP1 in facilitating the invasion and growth of tumor cells in LUAD. Nevertheless, further validation can be achieved by conducting *in vivo* experiments.

## 1 Introduction

Lung cancer is a highly malignant tumor with a high diagnostic frequency and ranks first in cancer-related deaths worldwide (1). Among the several histologic types of lung cancer, lung adenocarcinoma account for the highest percentage (2, 3). Over the past decades, significant progress has been made in exploring the molecular mechanism of LUAD progression, leading to the development of precision therapeutics such as tyrosine-kinase inhibitors (TKIs) (4). With the advent of molecular profiling, it has become clear that lung adenocarcinoma is a genetically heterogeneous disease, characterized by a range of driver mutations and alterations that are amenable to targeted therapy (5). Besides, mounting evidence has indicated a significant association between m6A regulators and malignant neoplasms (6). For example, the significant correlation between downregulation of METTL14 in liver cancer and tumor metastasis has been observed (7). Several prior studies have identified abnormal expression patterns of m6A regulators in LUAD as well (8). Molecular targeting of lung adenocarcinoma involves the use of drugs or other agents that specifically target the genetic alterations that are present in the cancer cells, with the aim of achieving more effective and less toxic treatments (9). Oncogenic KRAS is a prominent driver of lung adenocarcinoma (LUAD), which has yet to be effectively targeted by therapeutics. One study has presented evidence that the SLC7A11/glutathione axis demonstrates metabolic synthetic lethality with oncogenic KRAS. Research has demonstrated that LUAD cells harboring KRAS mutations are sensitive to SLC7A11 inhibition, suggesting possible therapeutic avenues for this presently untreatable condition (10). This approach has revolutionized the management of lung adenocarcinoma, leading to improved outcomes for patients with specific molecular subtypes of the disease. However, a considerable proportion of LUAD patients still experience poor prognosis due to innate or acquired resistance to targeted therapy (11). For example, Tyrosine kinase inhibitors (TKIs) targeting sensitizing mutations in the epidermal growth factor receptor (EGFR) gene constitute a vital cornerstone of non-small cell lung cancer management. Despite the outstanding disease control obtained through primary EGFR TKI therapy, the development of acquired resistance is pervasive and represents a major obstacle (12). Immunotherapy provides a novel approach to the management of LUAD patients (13). In recent years, immunotherapy has emerged as a promising treatment option for lung adenocarcinoma (14). Immunotherapy works by activating the body’s immune system to recognize and attack cancer cells. It does this by targeting specific proteins on the surface of cancer cells, called checkpoint proteins, that can inhibit immune cell activity (15). The two main types of immunotherapies used to treat lung adenocarcinoma are immune checkpoint inhibitors and adoptive cell therapy (16). Immune checkpoint inhibitors block checkpoint proteins on cancer cells, allowing immune cells to attack the cancer. Adoptive cell therapy involves removing immune cells from a patient’s body, genetically modifying them to recognize and attack cancer cells, and then infusing them back into the patient (17). Immunotherapy has shown promising results in treating lung adenocarcinoma, particularly in patients whose cancer has spread and is no longer responding to traditional treatments. However, it is not effective for all patients, and there can be significant side effects (18). Further research is needed to determine which patients will benefit most from immunotherapy and how to minimize side effects.

Cancer initiation, progression and immigration incur a range of dynamic alterations in host tissues, bringing about a complex tumor stroma, illustrated as the tumor microenvironment (TME) (19). The evolution and homeostasis of the TME largely depend on an intimate communication within and across several cellular compartments, including malignant, stromal, and immune cells. Among them, cancer-associated fibroblasts (CAFs) are the principal component of stromal cells and release inflammatory, growth factors, and extracellular matrix, accelerating tumor proliferation and contributing to therapy resistance (20). CAFs can promote progression of malignant cells by serving TME crucial nutrients, such as alanine and lipoids (21). Besides, accumulating evidence have confirmed that CAFs are significantly correlated with several cancers, such as breast cancer, gastrointestinal cancer and lung cancer (22–24). In-depth research on the crosstalk between CAFs and other TME cells could provide new insights into subsequent targeted therapy.

Despite significant efforts to study CAFs in LUAD, comprehensive characterization and prediction of immunotherapy response are lacking. Herein, transcriptome and single-cell RNA-sequencing (scRNA-seq) data from public database were collected and further processed. Distinguished CAFs subclusters were obtained and based on which a risk signature was established for LUAD. The signature’s independent prognostic prediction values were validated by several methods. A novel nomogram integrating the risk signature and clinicopathological features was constructed to facilitate the clinical application of CAF in LUAD. The risk signature, along with the novel nomogram, has the potential to enable more accurate patient stratification for LUAD and offer more precise prognostic predictions. Furthermore, the CAF-related signature was evaluated for immune landscape and responsiveness to immunotherapy, providing new insights into the management of LUAD and improving patient outcomes.

## 2 Methods

### 2.1 Data collection and processing

We obtained scRNA-seq data from the Gene Expression Omnibus (GEO) database (accession number GSE149655), which comprised four samples: two primary lung adenocarcinoma (LUAD) samples and two normal tissue samples. We filtered out single cells expressing fewer than 250 genes or those with any gene expressed in fewer than three cells. We also evaluated the percentage of mitochondria and rRNA using the PercentageFeatureSet function in the Seurat R package (25, 26). This resulted in a total of 12,554 cells for further analysis.

We collected transcriptome data, copy number variants (CNV), single-nucleotide variants (SNV), and corresponding clinical data of LUAD from The Cancer Genome Atlas (TCGA) database. We excluded samples lacking survival data or outcome status and included 500 tumor samples and 59 normal samples in the analysis. We also utilized two external validation cohorts: GSE72094 cohort with 398 samples and GSE26939 cohort with 115 samples after removing samples without follow-up.

From the literature, we identified ten cancer-associated pathways (HIPPO, Cell Cycle, MYC, NRF1, NOTCH, PI3K, RAS, TP53, TGF-Beta, and WNT) and analyzed their gene expression profiles in our dataset (27).

### 2.2 CAF definition

The Seurat package was used to re-analyze the scRNA-seq data of LUAD (28), with the aim of systematically characterizing the CAF signature. Firstly, expressed genes were log-normalized after removing cells with below 250 or over 6000 expressed genes. Then, the FindIntegrationAnchors function was employed to remove batch effects for the four samples. Non-linear dimensional reduction was performed using the uniform manifold approximation and projection method, with a resolution of 0.2 and 30 principal components selected. Subsequently, single cells were clustered into different subgroups using the FindNeighbors and FindClusters (dim = 30 and resolution =0.2) functions. UMAP dimensional reduction was performed using the RunUMAP function. Fibroblasts were annotated based on four marker genes, including FAP, PDGFRB, ACTA2, and NOTCH3. The fibroblasts were re-clustered using the same algorithm of FindClusters and FindNeighbors functions. Marker genes for each CAF cluster were defined with the FindAllMarkers function by comparing different clusters with minpct = 0.35, logFC =0.5, and adjust p-value<0.05. We also used the CopyKAT R package (29) to analyze the CNV characteristics of CAFs clusters and distinguish between tumor cells and normal ones. Finally, we performed Kyoto Encyclopedia of Genes and Genomes (KEGG) enrichment analysis on the marker genes using the clusterProfiler package (30).

### 2.3 Hub genes of CAF identification

Firstly, the limma package (31, 32) was used to identify differentially expressed genes (DEGs) between normal and tumor tissue based on |log2(FoldChange)|>1 and a false discovery rate (FDR)<0.05. Then, correlations between CAF clusters and DEGs were evaluated, followed by the identification of key CAF-related genes with p<0.01 and cor>0.4. To identify prognosis-related genes, univariate Cox regression analysis was conducted using the survival package (33). To reduce the number of genes, the least absolute shrinkage and selection operator (lasso) was performed (34–36). Multivariate Cox regression analysis was conducted using the stepwise regression method to establish a CAF-based risk signature, which was calculated using the formula: 0.123*CLEC3B+0.114*EXO1 + 0.103*CCNB1+-0.177*CD302. Patients were classified into low- and high-risk groups using zero-mean normalization. The predictive value of the risk signature was evaluated using receiver operating characteristic curve (ROC) analysis with the timeROC package (37, 38).

### 2.4 A novel nomogram constructed based on the risk signature

Following the univariate and multivariate Cox regression analysis based on the risk signature and clinicopathological features (39), we constructed a novel nomogram to predict the prognosis of LUAD using variables with p<0.05 in the multivariate Cox model. We evaluated the predictive accuracy of the model by generating a calibration curve.

### 2.5 Immune landscape analysis

We comprehensively assessed the correlation between the risk signature and the tumor immune microenvironment (TIME) using several algorithms, including CIBERSORT, EPIC, MCPCOUNTER, and TIMER. The “estimate” R package was used to calculate stromal scores, immune scores, and estimate scores (stromal scores + immune scores) to evaluate differences in the tumor microenvironment of patients. Additionally, we estimated the proportions of 22 immune cell subtypes using the CIBERSORT algorithm based on the TCGA cohort.

### 2.6 Responsiveness to immunotherapy

Anti-PD-1 or anti-PD-L1 checkpoint inhibition therapy has gained increasing attention as a crucial component of immunotherapy. To evaluate the performance of the risk signature in predicting responsiveness to immunotherapy (immune checkpoint blocks), we collected transcriptomic data as well as corresponding clinical data from patients who received anti-PD-L1 therapy from the IMvigor210 cohort. We also downloaded transcriptomic data from the GSE78220 cohort, which included melanoma patients who received anti-PD-1 checkpoint inhibition therapy before treatment.

### 2.7 Cell lines culture of lung adenocarcinoma cells and cell transfection

All patients conferred their informed consent before being enrolled in the study. Sample collections were conducted following procedures approved by the Ethics Committee of Jiangsu Province People’s Hospital (2019-SR-156). Lung adenocarcinoma cell lines including A549 and H1299 cells was purchased from ATCC. All cells were cultured using Ham’s F-12K and RPMI 1640 medium (Gibco, USA), supplemented with 10% FBS (HyClone Sera, USA) and 1% penicillin‐streptomycin (Sangon Biotech, China), and maintained in an atmosphere containing 5% CO2 at 37°C. The EXO1 siRNA expression vector and scrambled siRNA nontarget control were obtained from Genewiz (China). Plasmids were transfected using Lipofectamine 3000 (Thermo Scientific, USA), as per the manufacturer’s protocols (40).

### 2.8 RNA extraction and quantitative real-time polymerase chain reaction

Total RNA was extracted from the cell lines using TRIzol in accordance with the manufacturer’s instructions (15596018, Thermo). Subsequently, cDNA was synthesized utilizing the PrimeScript TMRT kit (R232-01, Vazyme). Real-time polymerase chain reaction (RT-PCR) was performed using SYBR Green Master Mix (Q111-02, Vazyme). The expression levels of each mRNA were standardized to the level of mRNA GAPDH, and the quantification of expression levels was executed using the 2^–ΔΔCT^ method (41).

### 2.9 Cell counting kit-8 assay and EdU

The suspension of cells was seeded in 96-well plates at a density of 5×10^3^ cells per well. After adding 10 μl of CCK-8 labeling agent (A311-01, Vazyme) to each well, the plate was incubated for 2 hours in the dark at 37°C. Cell viability was evaluated by measuring absorbance at 450 nm at 0, 24, 48, 72, and 96 hours using an enzyme-labeled meter (A33978, Thermo). The experiment was performed using a 96-well plate with 2×10^4^ treated cells in each well, after the cells had adhered to the wall. The 5-Ethynyl-2’-deoxyuridine (EdU) assay was performed according to the manufacturer’s instructions (Ribobio, China), and cell proliferation was quantified using an inverted microscope.

### 2.10 Wound-healing assay and transwell assay

The transfected cells were seeded in 6-well plates and cultured in a cell incubator until they reached 95% confluence. Each well was gently scraped using a sterile 200 μl plastic pipette tip, and any unattached cells and debris were rinsed twice with PBS. The breadth of the scratch wounds was measured using the Image J program, and photographs were taken at 0 h and 48 h. For the cell invasion and migration experiments, treated A549 and H1299 cells (2×10^4^) were incubated in the upper chamber of 24-well plates for 48 hours. The top surface of the plate was either precoated with matrigel solution (BD Biosciences, USA) or left untreated to assess the cells’ ability to invade and migrate. After removing the cells from the top surface, the remaining cells on the bottom layer were fixed with 4% paraformaldehyde and stained with 0.1% crystal violet (Solarbio, China).

### 2.11 Statistical analysis

All statistical analyses were performed using R software (version 4.1.0). Wilcoxon test was used for comparing two groups, while Spearman or Pearson correlation was used for correlation matrices. The Log-rank test was used to assess survival differences through K-M curves, where statistical significance was defined as p-value < 0.05.

## 3 Results

### 3.1 CAF clustering and screening in scRNA samples

**Figure 1:**
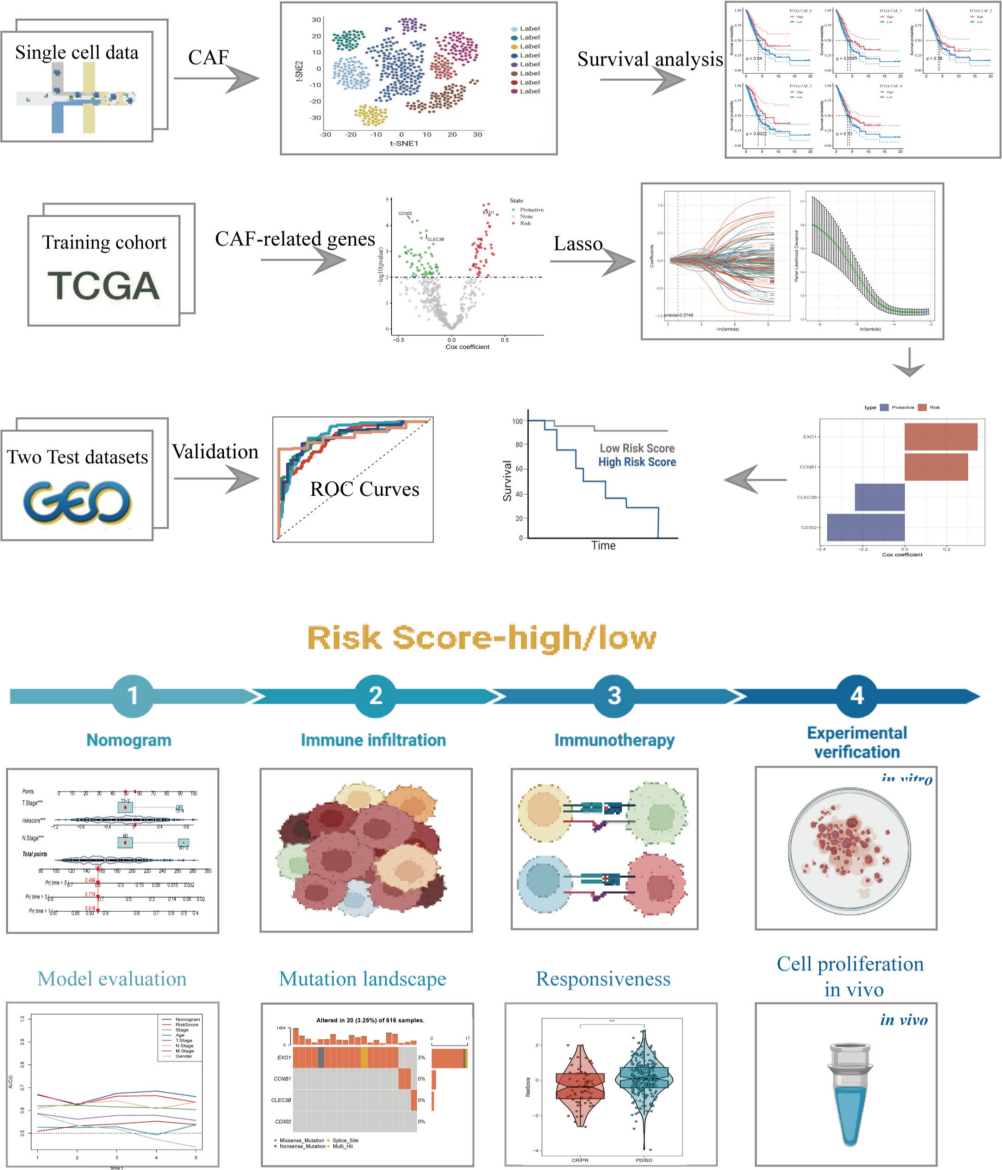
The flow chart of this study.

**Figure 2:**
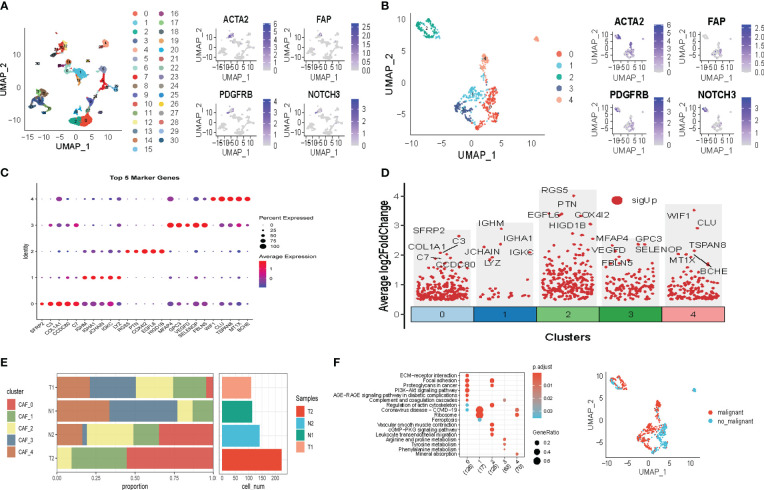
The identification of CAF clusters according to scRNA-data of LUAD patients. **(A)** Umap plots of distribution of 31 clusters and fibroblasts-based marker genes expression. **(B)** Umap plots of distributions of five fibroblasts after clustering. **(C)** Bubble diagram of the top5 marker gene expression of subgroups. **(D)** Volcano plot of the top5 marker gene expression of subgroups. **(E)** Subgroups in cancer and adjacent tissue and proportion as well as cell number calculation. **(F)** KEGG analysis of five fibroblasts subgroups. **(G)** Umap distribution of malignant and non-malignant cells predicted by copycat package.

The flow chart of our study is depicted in **Figure 1**. Following initial screening, a total of 12,554 cells were obtained from scRNA-seq data. Detailed results of data preprocessing are presented in **Figure S1**. Firstly, after performing log-normalization and dimensionality reduction, we identified 31 subpopulations (**Figure 2A**). Using four marker genes (FAP, FDGFRB, ACTA2, and NOTCH3), we further identified five CAF populations, as shown in **Figure 2B**. Cells collected from the four CAF populations were then separated for clustering and dimensionality reduction in subsequent research. Utilizing the same clustering algorithm, we discovered five clusters, as depicted in **Figure 2B**. Moreover, after performing the R package ‘FindVariableFeatures’, we obtained 756 DEGs from the five CAF clusters. The top 5 DEGs, which were characterized as CAF cluster marker genes, are exhibited in **Figures 2C, D**. Histograms illustrating the proportion of the five clusters in each cohort are shown in **Figure 2E**. Furthermore, KEGG analysis revealed that these DEGs were enriched in divergent pathways such as ECM-receptor interaction, focal adhesion, proteoglycans in cancer, PI3K-Akt signaling pathway, and others, as presented in **Figure 2F**. Finally, the distribution of tumor and normal cells based on the five CAF clusters according to the CNV characteristics is shown in **Figure 2G**.

### 3.2 The analysis of cancer-related pathways in CAF

**Figure 3:**
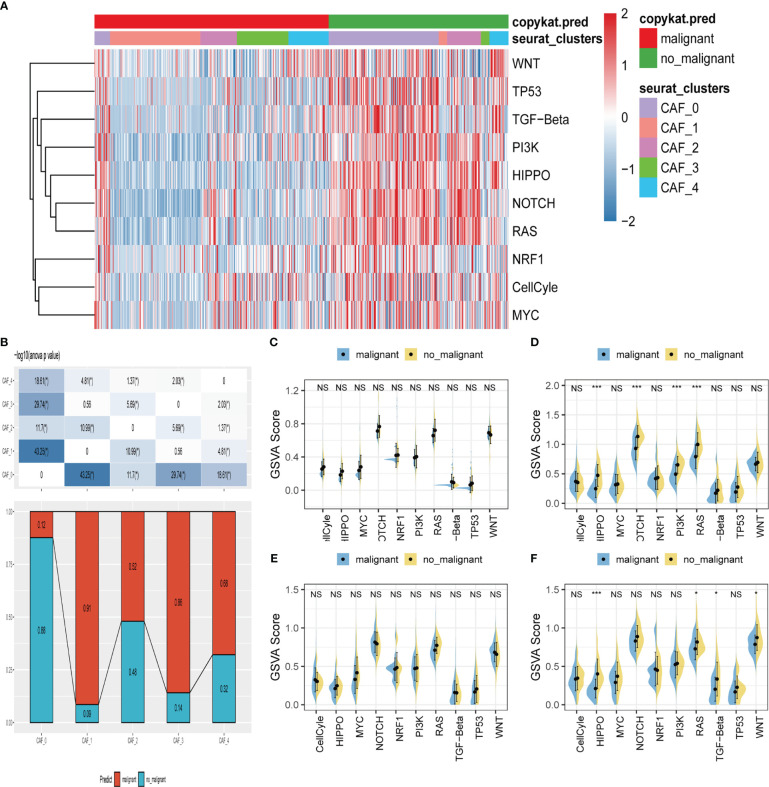
The characteristics of tumor-associated pathways in CAF clusters. **(A)** Heatmap of 10 tumor-associated pathways enriched in CAF cells. **(B)** Comparison between each cluster based on proportions of malignant and non-malignant cells. Comparison of each pathway between malignant and non-malignant cells based on GSVA score in CAF_0 (**Figure S2A**), CAF_1 **(C)**, CAF_2 **(D)** CAF_3 **(E)** CAF_4 **(F)**. (Wilcox. Test, *P < 0.05; ***P < 0.001; ns, not significant.).

We explored the characteristics of ten tumor-related pathways in the five CAF clusters to elucidate the correlations between tumor progression and the CAF clusters. GSVA was employed to investigate the underlying mechanisms involved in the progression and prognostication of LUAD. GSVA scores of those pathways were calculated based on different CAF clusters, and the results are presented in **Figure 3A**. As shown in **Figure 3B**, the percentage of normal cells in CAF_0 cluster was the highest, while the ratio of malignant cells from CAF_1 was significantly higher than that in the others. Significant differences were only identified among the CAF_0, CAF_2, and CAF_4. Furthermore, GSVA scores were analyzed based on the ten tumor-related pathways between normal and malignant cells in each CAF cluster, with slight differences observed in CAF_2 and CAF_4 (**Figures 3C–F**). (The results of GSVA score analysis in CAF_0 is shown in **Figure S2**).

Moreover, to explore the correlations between the CAF clusters and crucial clinicopathologic features, we analyzed the ssGSEA score of the marker genes (the top 5 DEGs referred to in **Figures 2C, D**) of each CAF cluster according to the TCGA cohort. The results showed that tumor samples had a significantly higher score compared with normal ones in each cluster (**Figure S2B**). Using the survminer R package, LUAD samples of TCGA cohort were divided into low-and-high CAF score groups based on the optimal cut-off value. The samples in the low-CAF score group had a significantly worse prognosis in CAF_0, CAF_1, and CAF_3. Although no significant differences were observed in the other two clusters, there was a trend that patients in high-CAF score groups shared a better prognosis (**Figure S2C**). Furthermore, other clinicopathologic features, including T. stage, N. stage, M. stage, and stage, were included in the analysis. Only slight differences were observed between low-and-high CAF group patients (**Figure S3**). However, patients in high-CAF groups tended to share favorable clinicopathologic features.

### 3.3 Hub genes identification and risk signature construction

**Figure 4:**
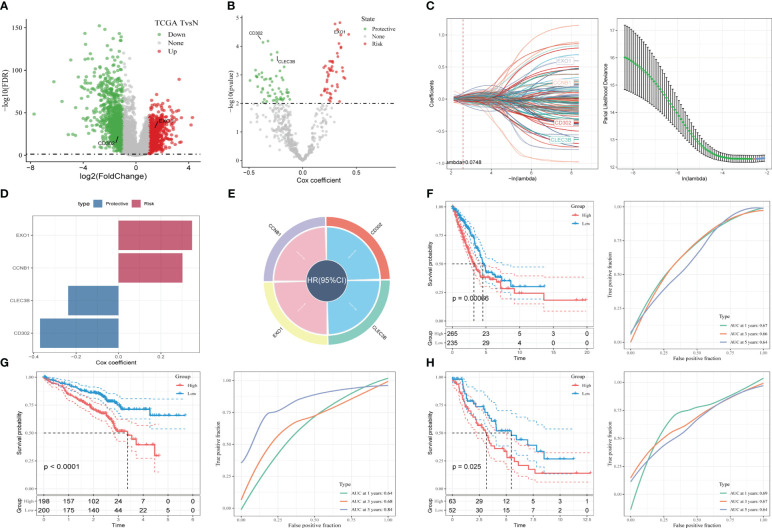
A novel risk signature constructed based on several CAF-related genes. **(A)** Volcano plot of differentially expressed genes between tumor and normal samples in TCGA cohort. **(B)** Volcano plot of prognosis-correlated genes obtained by univariate Cox regression analysis. **(C)** Each independent variable’s trajectory and distributions for the lambda. **(D-E)** The multivariate Cox coefficients for each gene in the risk signature. **(F)** K-M and ROC curves of the risk signature in TCGA cohort. **(G)** K-M and ROC curves of the risk signature in GSE72094 cohort. **(H)** K-M and ROC curves of the risk signature in GSE26939 cohort.

To construct a risk signature, we first screened for DEGs between normal and tumor tissues. A total of 1731 DEGs were obtained, with 725 up-regulated and 1006 down-regulated (**Figure 4A**). Out of these, 492 genes were significantly correlated with the prognosis-related CAF clusters. Using univariate Cox regression analysis, we further evaluated the prognosis of each gene, identifying 49 genes related to risk factors and 62 genes exhibiting protective values (**Figure 4B**). To reduce the number of genes, we conducted Lasso Cox regression analysis, resulting in 4 genes with lambda=0.074 (**Figure 4C**). Finally, we used the stepwise regression method to construct the risk signature after multivariate Cox regression analysis. The signature consists of 4 genes (**Figures 4D, E**), namely Exonuclease 1 (EXO1), Cyclin B1 (CCNB1), C-Type Lectin Domain Family 3 Member B (CLEC3B), and Type I C-type lectin receptor CD302. The final signature formula is as follows: RiskScore = -0.123*CLEC3B+0.114*EXO1 + 0.103*CCNB1+-0.177*CD302. Using z-mean normalization, we calculated the risk score for each sample, dividing patients into high and low-risk groups. Kaplan-Meier survival analysis showed that low-risk patients had significantly better survival outcomes compared to high-risk patients, not only in the TCGA cohort (**Figure 4F**) but also in the GSE72094 (**Figure 4G**) and GSE26939 (**Figure 4H**) cohorts. Additionally, based on the TCGA and GEO cohorts, the AUC values of the signature for 1-3-5-year survival were satisfying, indicating the model’s excellent predictive power (**Figures 4F-H**). We also presented the distribution of risk score, patient survival status, and expression of hub genes in the TCGA cohort in **Figure S3A**. Similarly, the results of GSE72094 and GSE26939 were shown in **Figures S3B-C**.

### 3.4 Recognition of independent risk factors and development of nomogram

**Figure 5:**
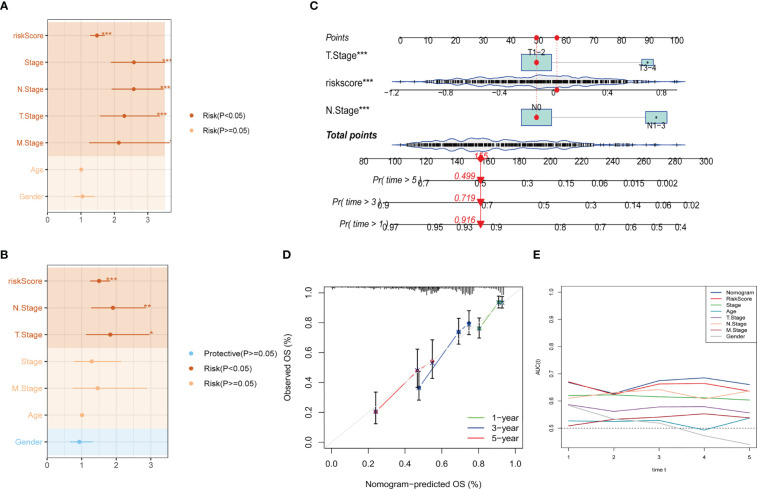
Development of a novel nomogram integrating the risk signature and several clinicopathologic features. **(A)** Results of univariate Cox regression analysis based on risk score and clinicopathologic features. **(B)** Results of multivariate Cox regression analysis based on risk score and clinicopathologic features. **(C)** Construction of the nomogram integrating the risk score and clinical stage. **(D)** Calibration curves for 1, 3, and 5 years of nomogram. **(E)** Evaluation of predictive capacity of nomogram and clinicopathologic features by time-ROC analysis. (*P < 0.05; **P < 0.01; ***P < 0.001).

To construct a more accurate predictive model, we integrated the risk score with clinicopathological characteristics using both univariate and multivariate Cox regression analyses. The multivariate analysis demonstrated that the risk signature was the most significant independent prognostic factor for lung adenocarcinoma (p-value < 0.001), followed by N-stage (**Figures 5A, B**). Accordingly, we developed a novel nomogram incorporating T-stage, N-stage, and risk score (**Figure 5C**), which demonstrated strong predictive power for actual survival outcomes according to calibration plot analysis (**Figure 5D**). TimeROC analysis in the TCGA cohort confirmed that the AUC of the nomogram and risk score exceeded that of other indicators (**Figure 5E**).

### 3.5 Correlations between the risk signature and clinicopathologic features in LUAD patients

After examining the clinicopathologic features (Age, Gender, Stage, T-stage, N-stage, and M-stage) between high- and low-risk groups, we found that gender, T-stage, N-stage, and stage were significantly associated with the risk signature (**Figure S4A**). These findings were consistent with previous studies that identified gender as a risk factor for LUAD, with males being more likely to be in the high-risk group (**Figure S4B**). Moreover, patients in the high-risk group tended to have more advanced clinical stages (**Figures S4C-E**).

### 3.6 Tumor mutation analysis

**Figure 6:**
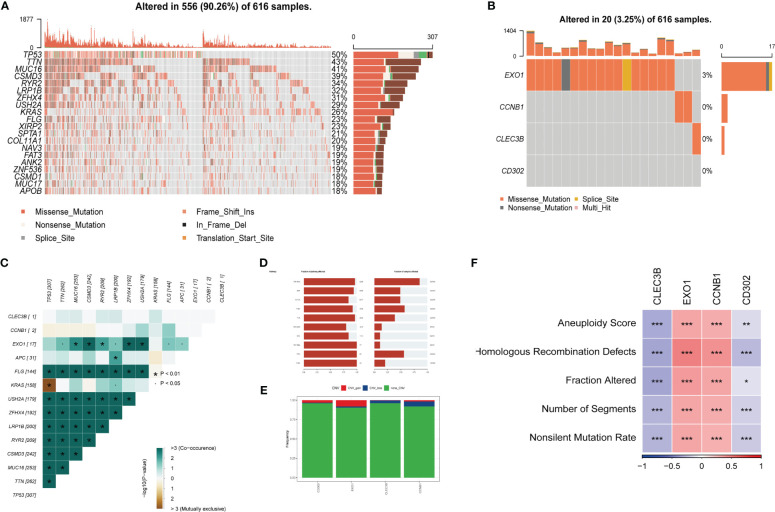
Tumor mutations analysis (TMB) **(A)** The landscape of mutations based on the TCGA cohort. **(B)** Waterfall diagram displaying SNV mutations of four key genes. **(C)** Mutual exclusion and collinearity analysis of the four genes and the 10 most mutated genes in tumors. **(D)** The proportions of 10 tumor-related pathways were depicted. **(E)** CNV mutations (gain, loss, none) of four key genes. **(F)**  Correlation heatmap of six key genes with Homologous Recombination Defects, Aneuploidy Score, Number of Segments, Fraction Altered, and Nonsilent Mutation Rate. (*P < 0.05; **P < 0.01; ***P < 0.001).

Exploring SNV mutations in lung adenocarcinoma based on the TCGA cohort to investigate the SNV mutations in lung adenocarcinoma, the top 20 genes with the highest mutation frequency were analyzed based on the TCGA cohort, as shown in **Figure 6A**. Subsequently, the SNV mutations of the four genes in the risk signature were examined. As displayed in **Figure 6B**, EXO1 had the highest mutation frequency, with Missense-Mutation being the most common type of mutation. Conversely, no mutations were observed in CD302. Additionally, the probability of co-occurrence of the 10 most mutated genes and the risk genes (except for CD302) was assessed, and the results indicated a low likelihood of co-occurrence of mutations in these three genes. However, EXO1 was found to significantly co-occur with MUC16, CSMD3, RYR2, ZFHX4, and USH2A (**Figure 6C**). Further analysis revealed that only a few samples had loss/gain of CNV based on the four genes (**Figure 6E**). The fraction of the pathway affected by these risk genes was also calculated in the TCGA cohort (**Figure 6D**). Moreover, the relationships between the risk genes and several molecular signatures of LUAD were explored to demonstrate the links between the risk genes and LUAD. The results indicated that EXO1 and CCNB1 were positively correlated with molecular signatures such as Aneuploidy Score, Homologous Recombination Defects, and Fraction Altered, while CLEC3B and CD302 were negatively correlated with these signatures (**Figure 6F**).

### 3.7 Gene set enrichment analysis

**Figure 7:**
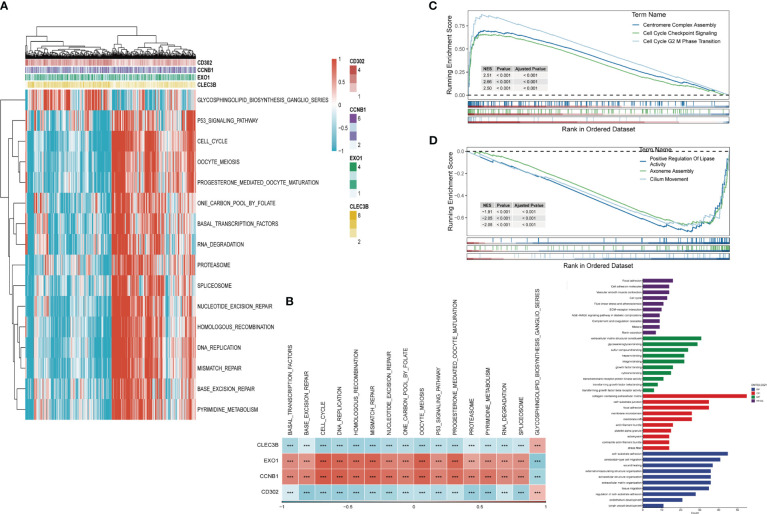
Gene Set Enrichment Analysis (GSEA) **(A)** Heatmap exhibiting enrichment score for key pathways based on the hub genes. **(B)** Gene-pathway correlation heatmap, **(C)** Pathways enriched in high-risk group. **(D)** Pathways enriched in low-risk group. **(E)** KEGG and GO analysis. (***P < 0.001).

Based on these four genes from the risk signature, Gene Set Enrichment Analysis was performed. The results showed that 16 pathways were significantly correlated with these four genes in total (**Figures 7A, B**), such as the p53 signaling pathway, cell cycle, and DNA replication. Similar to the results obtained previously, EXO1 and CCNB1 were positively correlated with these pathways, while CD302 and CLEC3B were negatively related to them. The GSEA score was estimated based on the high-and-low-risk subgroups (**Figures 7C, D**). Centromere Complex Assembly, Cell Cycle Checkpoint Signaling, and Cell Cycle G2 Phase Transition were significantly enriched in the high-risk group. In contrast, Positive Regulation of Lipase Activity, Axoneme Assembly, and Cilium Movement were significantly enriched in the low-risk group. Finally, the results of KEGG and GO analysis are shown in **Figure 7E**.

### 3.8 Landscape of immune infiltrations and relationship between risk genes and immunity

**Figure 8:**
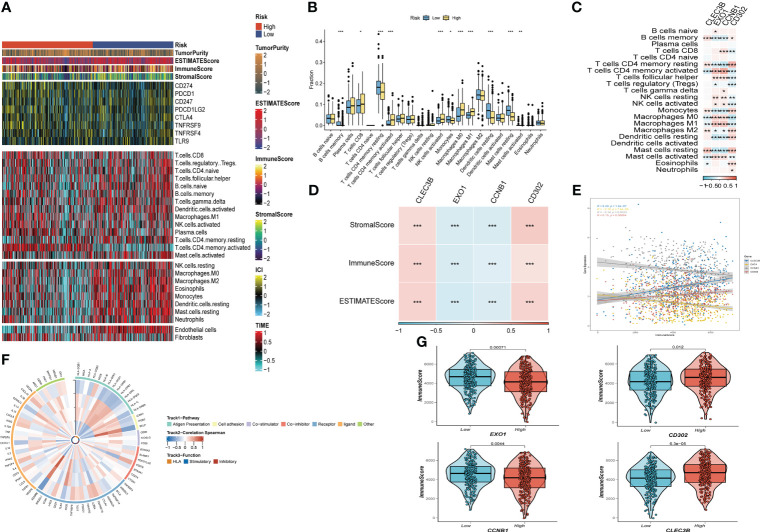
The immune infiltrations analysis **(A)** Heatmap of results on immune cells of tumor microenvironment (TME) in LUAD with multialgorithm, including existing data from platform TIMER and MCP-counter. TME-related scores were exhibited in the top bar. **(B)** Comparison of proportions of 22 immune-related cells between high-and-low-risk groups. **(C)** Correlations between four hub genes and 22 immune-related cells. **(D,E,G)** Correlations between the four hub genes and immune score, stromal score, estimate score. **(F)** The correlation analysis between four hub genes and 75 immune-associated genes. (*P < 0.05; **P < 0.01; ***P < 0.001).

After investigating the landscape of immune and stromal cell infiltrations in both low- and high-risk groups, we found that **Figure 8A** illustrates how patients in the low-risk group have higher proportions of immune and stromal cell infiltrations compared to those in the high-risk group. Moreover, using the CIBERSORT algorithm (42, 43), we calculated the immune cell proportions between the high- and low-risk groups (**Figure 8B**) and found that patients in the high-risk group significantly shared higher proportions of CD8 T cells, activated memory CD4 T cells, activated NK cells, Macrophages (M0), and Macrophages (M1). On the other hand, B cells, resting memory CD4 T cells, Monocytes, resting dendritic cells, and Activated mast cells were significantly enriched in the low-risk group.

We then explored the relationship between risk genes and immunity. Our results showed that EXO1 and CCNB1 had a significantly negative relationship with the majority of T cells, while CLEC3B and CD302 were remarkably correlated with Macrophages (**Figure 8C**). Additionally, correlation analysis presented that EXO1 and CCNB1 were negatively linked with Stromal Score, Immune Score, and ESTIMATE Score. In contrast, the other risk genes exhibited the opposite trend (**Figures 8D, E, G****)**. Finally, **Figure 8F** revealed the correlation between the four risk genes and the 75 immune-related genes.

### 3.9 Response to PD-L1 blockade immunotherapy based on risk signature

**Figure 9:**
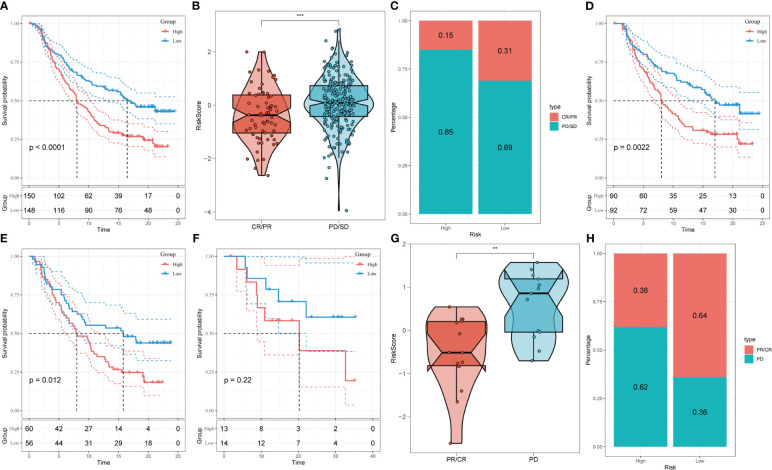
Prediction of responsiveness to immunotherapy using our signature based on public database. **(A)** Prognostic differences between risk subgroups in the IMvigor210 cohort. **(B)** Differences among immunotherapy responses based on risk scores in the IMvigor210 cohort. **(C)** Distribution of immunotherapy responses based on risk subgroups in the IMvigor210 cohort. **(D)** Prognostic differences between risk subgroups based on early stage (stage I-II) in the IMvigor210 cohort. **(E)** Prognostic differences between risk subgroups based on advanced patients (stage III-IV) in the IMvigor210 cohort. **(F)** Prognostic differences between risk subgroups in the GSE78220 cohort. **(G)** Differences among immunotherapy responses based on risk scores in the GSE78220 cohort. **(H)** Distribution of immunotherapy responses based on risk subgroups in the GSE78220 cohort. (**P < 0.01; ***P < 0.001).

We analyzed the response to PD-L1 blockade immunotherapy in the IMvigor210 and GSE78220 cohorts after assessing immune infiltrations. The 348 patients from the IMvigor210 cohort presented different responses to anti-PD-L1 receptor blockers, including stable disease (SD), partial response (PR), complete response (CR), and progressive disease (PD). We found that CR/PR patients had lower risk scores than SD/PD patients (**Figure 9B**). Additionally, in the low-risk group, the proportion of SD/PD patients was lower than that in the high-risk group (**Figure 9C**). Our analysis of the IMvigor210 cohort revealed that patients in the low-risk group had significantly better clinical outcomes than those in the high-risk group (**Figure 9A**). Furthermore, we identified significant survival differences between different risk groups not only in Stage I+II but also in Stage III+IV patients (**Figures 9D, E**).

To confirm our findings, we enrolled the GSE78220 cohort into further analysis. Corresponding with the results obtained in IMvigor210, PR/CR patients had lower risk scores and shared a lower percentage in the high-risk group (**Figures 9F–H**).

### 3.10 Validation of the tumor-related role of EXO1 in NSCLC

**Figure 10:**
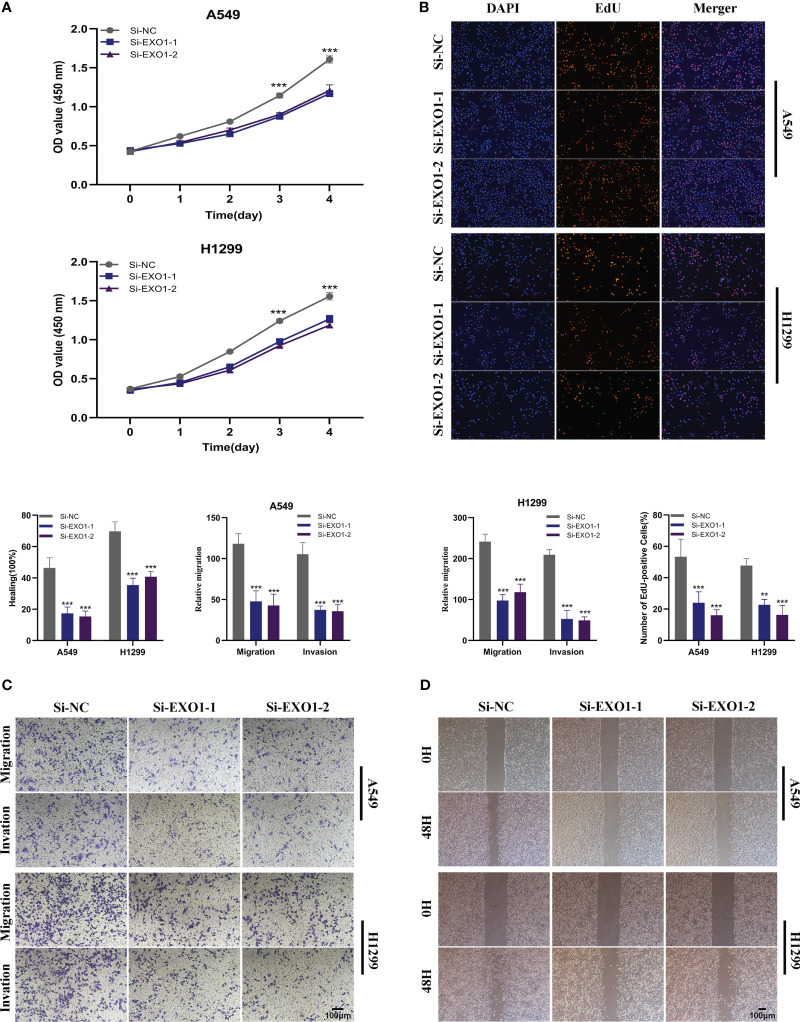
The role of EXO1 in LUAD. **(A)** CCK8 assay showed that, after EXO1 knockdown, the cells showed significant reduction in viability. **(B)** EdU staining assay indicated that downregulation of EXO1 expression repressed cell proliferation in LUAD cell lines. **(C)** Transwell assay showed that downregulation of EXO1 expression inhibited the migration and invasion capacity of LUAD cells. **(D)** Scratch-wound healing assay depicted that a significantly slower wound healing rate was observed in cells with a decreased expression of EXO1. To demonstrate the accuracy and reproducibility of the results, all experiments were repeated in two LUAD (A549, H1299) cell lines and all data were presented as the means ± SD of three independent experiments. **P < 0.01, ***P < 0.001.

In order to further elucidate the function of EXO1 in LUAD, we conducted *in vitro* research to scrutinize the function of EXO1 in LUAD cells. We gauged the degree of EXO1 expression after 24 hours of transfection *via* qRT-PCR to assess the efficacy of siRNA-mediated EXO1 knockdown in A549 and H1299 cell lines. As compared to the NC group, we observed a marked reduction in the expression of EXO1 in A549 and H1299 cells upon treatment with siRNA (Si-1 and Si-2) sequences (P < 0.001) (**Figures 10**). Correspondingly, the CCK-8 assay revealed that suppression of EXO1 significantly curbed the viability of A549 and H1299 cells as compared to control cells (**Figure 10A**). The findings of the EdU staining assay provided further evidence that inhibition of EXO1 expression impeded the proliferation of A549 and H1299 cells relative to the NC group (**Figure 10B**). This implies that EXO1 might play an indispensable role in the proliferation of LUAD cells. The transwell experiments confirmed that EXO1 knockdown considerably reduced the migration and invasion of A549 and H1299 cells (**Figure 10C**). The scratch-wound healing experiment also produced congruent results, wherein decreased EXO1 expression led to a noteworthy deceleration in the rate of wound healing in cells (**Figure 10D**). To ensure the accuracy and consistency of the results, all tests were performed in two LUAD cell lines (A549 and H1299), and all data were presented as means with standard deviations from three independent experiments. *P < 0.05, **P < 0.01, ***P < 0.001.

## 4 Discussion

With a growing understanding of the tumor microenvironment (TME), research focus has broadened from immune cells to other cellular components, such as cancer-associated fibroblasts (CAFs) (22). As a crucial component of the TME, CAFs have divergent functions, such as matrix remodeling and deposition, extensive reciprocal signaling interactions with infiltrating leukocytes and crosstalk with cancer cells (44). Cancer-associated fibroblasts (CAFs) present in primary and metastatic neoplasms are extremely adaptable, malleable, and robust cells that actively participate in the advancement of cancer by engaging in intricate cross-talk with other cellular entities in the tumor microenvironment (19). Within the microenvironment of cancer, stromal cells play a significant role, and among them, cancer-associated fibroblasts (CAFs) make up the largest portion and are closely linked to cancer progression. Additionally, the cancer stroma can promote tumor recurrence and contribute to therapeutic resistance, explaining why current anti-tumor therapeutic approaches often fail to eliminate malignancy (45). Therefore, investigating the tumor microenvironment (TME) with a focus on cancer-associated fibroblasts (CAFs) could not only enhance our understanding of their phenotypic diversity but also provide new insights into anti-tumor therapies. In this study, we utilized single-cell RNA sequencing (scRNA-seq) data to investigate the heterogeneity of CAFs and systematically identify and classify them in lung adenocarcinoma (LUAD). As a result, we identified five distinct CAF clusters that may play a critical role in regulating the TME’s biology. Furthermore, growing evidence suggests that a CAF-related gene signature can accurately predict the prognosis of LUAD patients (46, 47). Consistent with these findings, our results showed that three of the clusters had significant association with LUAD prognosis. By performing the GSVA analysis, a slight correlation was observed between the ten cancer-related pathways and the five clusters. The Hippo signaling was significantly enriched in no-malignant part in our data, and a recent study has revealed that Hippo signaling might work as a crucial tumor suppressor pathway, which may account for the prognostic value of CAF to some extent.

Next, we constructed a CAF-related risk signature using four genes based on the prognostic values of the three identified CAF clusters. The risk signature included two risk genes (EXO1 and CCNB1) and two protective genes (CLEC3B and CD302). To assess the accuracy of this signature, we validated it using external cohorts, including TCGA, GSE72094, and GSE26939, and obtained favorable results. Notably, EXO1, a crucial nuclease associated with the mismatch repair system, was among the genes included in the risk signature. Dysregulation of this gene has been linked to proliferation, migration, and invasion in LUAD (48). Besides, exonuclease 1 (EXO1) constitutes a plausible prognostic biomarker and exhibits significant correlations with immune infiltrates in lung adenocarcinoma (49). Moreover, one research suggested that the high expression of EXO1 was significantly correlated with aneuploidy, promoting tumor invasion in LUAD (50). Cell cycle-promoted factor CCNB1 can be targeted by VPS33B *via* c-Myc/p53/miR-192-3p to modulate the pathogenesis of non-small cell lung cancer (NSCLC) (51). In addition, CCNB1 can be directly targeted by microRNA-718, suppressing tumor immigration NSCLC (52). CLEC3B and CD302 have been verified downregulated in lung cancer and having the diagnostic and prognostic values in lung cancer (53, 54). The patients were then classified into high- and low-risk groups based on the median risk score, and subsequent analysis demonstrated that the low-risk group had a significantly better prognosis than the high-risk group. Furthermore, univariate and multivariate Cox regression analyses confirmed that the risk score was an independent predictor of overall survival (OS). We developed a nomogram based on the risk signature, which demonstrated a high degree of consistency between predicted and observed results regarding the OS of LUAD patients. In conclusion, our findings indicate that the risk signature we constructed can accurately predict the prognosis of LUAD patients. With the risk signature and novel nomogram, early and accurate diagnosis of LUAD could be achieved and patients will be stratified more appropriately.

Considering the fact that precise therapy for lung cancer rely on comprehensive genomic analyses (55), the mutation profile of LUAD patients based on TCGA cohort were depicted, which reflected the high frequency of mutations of LUAD. The tumor mutation burden was further performed based on our risk signature. Among them, EXO1 was the only gene observed with mutations in our data. Besides, EXO1 was identified co-occurring with several highly mutated genes, including MUC16, CSMD3, RYR2, ZFHX4, and USH2A. Additionally, EXO1 was found positively correlated with several molecular signatures, such as aneuploidy, fraction altered, and so on. One study has indicated that the hyper-excision of DNA triggered by a deficiency in MLH1, *via* exonuclease 1, stimulates the cGAS-STING pathway, thereby facilitating the migration of tumors (56). As reported, high level of aneuploidy is related to lung cancer progression (57). Taken as a whole, we can infer that high expression level of EXO1 might prospect unfavorable clinical outcomes, and further endeavor on EXO1 research might promote the development of precision therapeutics.

To elucidate the divergent pathways that the genes involved in the signature enriched, Gene Set Enrichment Analysis (GSEA) was conducted. The results revealed that risk genes (EXO1 and CCNB1) were positively linked with several pathways, including p53 signaling pathway, cell cycle, DNA replication, etc. The protective genes (CD302 and CLEC3B), however, were positively associated with only one pathway-glycosphingolipid_biosynthesis_ganglio_series. Accumulating evidence has confirmed that several crucial molecules could propel the proliferation, migration, and invasion *via* p53 signaling pathway and DNA replication in LUAD (58–60). Moreover, high expression of EXO1 and CCNB1 was identified significantly correlated with p53 signaling pathway, cell cycle, and DNA replication (61, 62). Then, GSEA was performed according to high-and-low-risk groups. The high-risk group was remarkably enriched in centromere complex assembly, cell cycle checkpoint signaling, and cell cycle G2 M phase transition, which have been confirmed significantly correlated with progression in LUAD (63–65).

Far from only aggregates of malignant cells, tumors are well-organized complex ecosystems (66). Consisting of distinct immune cell populations in tumor islands, the TIME is dramatically correlated with the antitumor immunological state of the TME (67). The TIME have long been identified substantially associated with tumor progression, recurrence and metastasis (68). To further understand the implications of our risk signature, we assessed the immune infiltration state using various algorithms. Our results demonstrated that the low-risk group had a higher level of immune cell infiltration, suggesting that this group was more likely to establish a “hot” tumor state that could accelerate the immune system to inhibit tumor progression. In contrast, the high-risk group had higher levels of M0 and M1 macrophages. A recent study has revealed that M0 to M2 polarization is linked to the immune suppression (69). We also investigated the correlations between the four genes included in the risk signature and the 22 immune infiltration cells. Our results showed that EXO1 was positively linked with various types of T cells, suggesting that it could be a potential target for immunotherapy. Moreover, the risk genes (EXO1 and CCNB1) were found to be negatively associated with stromal score, immune score, and estimate score. Despite the emergence of immunotherapy, a significant number of LUAD patients still experience this highly malignant tumor due to innate or acquired resistance to such therapies (70). Therefore, it is crucial to identify patients who are likely to benefit from immunotherapy. Using the IMvigor210 and GSE78220 cohorts, we found that our risk signature could effectively classify patients who were more likely to benefit from immunotherapies. In summary, our risk signature based on CAFs can independently predict the prognosis of LUAD patients and predict their responsiveness to immunotherapy.

Nevertheless, there are some limitations in our study that need to be addressed. Firstly, the risk signature was established using retrospective data from public databases. Therefore, more prospective and multi-center LUAD cohorts are required to eliminate bias. Secondly, we only predicted the responsiveness to anti-PD-L1 immunotherapy using our risk signature. Further research is necessary to evaluate the potential of our risk signature to predict the response to other precision therapies in the future.

## 5 Conclusion

In our study, we extensively investigated the CAF populations in LUAD and identified five CAF clusters with distinct characteristics. Three of these clusters were found to be significantly associated with LUAD prognosis and were used to establish a prognostic risk signature consisting of 4 genes based on the CAFs. Furthermore, we developed a novel nomogram that combined the risk signature and clinicopathological characteristics, which performed exceptionally well in predicting the clinical outcome of patients with LUAD. Our risk signature was also observed to be associated with tumor mutations and immune landscape. Additionally, our results indicated that the risk signature is suitable for predicting the responsiveness of LUAD patients to immunotherapy targeting PD-L1 blockade.

## 6 Ethical approval and consent to participate

All human experiments in this study have been approved by the Ethics Committee of the First Affiliated Hospital of Nanjing Medical University. All subjects gave their informed consent for inclusion before they participated in the study. The study was conducted in accordance with the Declaration of Helsinki, and approved by the Ethics Committee of the First Affiliated Hospital of Nanjing Medical University (protocol code No.2019-SR-156; 12 June 2019).

## Data availability statement

The datasets presented in this study can be found in online repositories. The names of the repository/repositories and accession number(s) can be found within the article/**Supplementary Materials**.

## Ethics statement

This study was approved by the Ethics Committee of Jiangsu Province People’s Hospital (2019-SR-156), written informed consent was obtained from the patients/participants.

## Author contributions

QR, PZ, HL and YY contributed conception and design of the study. YF, HaC and XZ finished the dada collection. HuC performed the statistical analysis. QR wrote the first draft of the manuscript. HL and YY revised the manuscript. ZX, HuC and YY gave the final approval of the version to be submitted.

## Supplementary material

Supplementary Figure 1 The details of re-process of scRNA-seq data of LUAD. **(A)** The relationship between the amount of mRNA/UMI and mitochondrial genes, the relationship between the amount of mRNA and UMI. **(B)** The relationship among UMI, mRNA, mitochondrial content, and rRNA of each sample before filtering. **(C)** Violin plots exhibited the expression of CAF-associated marker genes before clustering. **(D)** Violin plots displayed the expression of CAF-associated marker genes after clustering.

Supplementary Figure 2 **(A)** Comparison of each pathway between malignant and non-malignant cells based on GSVA score in CAF_0. **(B)** Comparison of five CAF scores in malignant and non-malignant tissues. **(C)** K-M curves of high-and-low CAF-score groups in the five clusters.

Supplementary Figure 3 Validation of the signature’s capacity in prognosis prediction **(A)** Distribution of risk scores and survival status in TCGA cohort. **(B)** Distribution of risk scores and survival status in GSE72094 cohort. **(C)** Distribution of risk scores and survival status in GSE26939.

Supplementary Figure 4 The relationship between risk score and several clinicopathologic features. **(A)** The landscape of differences between high-and-low-risk subgroups. The differences between high-and-low-risk groups based on Gender **(B)**, Stage **(C)**, T-stage **(D)**, N-stage **(E)**.
