# Supplementary material for: A novel signature predicts prognosis and immunotherapy in lung adenocarcinoma based on cancer-associated fibroblasts
Source: Front Immunol. 2023 May 31;14:1201573. doi: 10.3389/fimmu.2023.1201573 (PMC10264584; doi:10.3389/fimmu.2023.1201573)
Supplement: Supplementary file 1 [file DataSheet_1.docx]

<https://www.jianguoyun.com/p/DQFHkEUQjdemCxiSlv8EIAA>
