# Supplementary figures and images for: A novel signature predicts prognosis and immunotherapy in lung adenocarcinoma based on cancer-associated fibroblasts
Source: Front Immunol. 2023 May 31;14:1201573. doi: 10.3389/fimmu.2023.1201573 (PMC10264584; doi:10.3389/fimmu.2023.1201573)

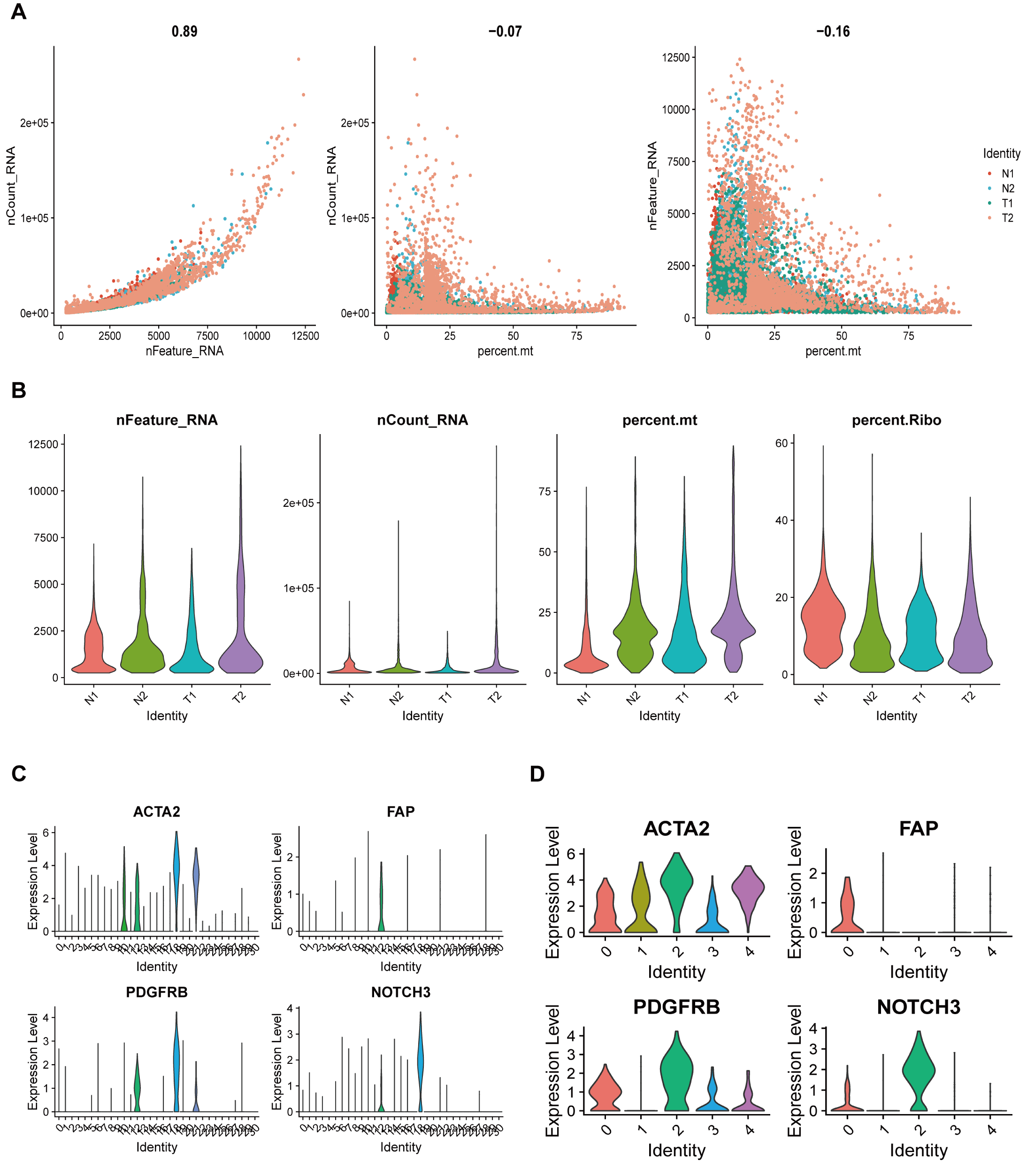

Supplement: Supplementary Figure 1 — The details of re-process of scRNA-seq data of LUAD. (A) The relationship between the amount of mRNA/UMI and mitochondrial genes, the relationship between the amount of mRNA and UMI. (B) The relationship among UMI, mRNA, mitochondrial content, and rRNA of each sample before filtering. (C) Violin plots exhibited the expression of CAF-associated marker genes before clustering. (D) Violin plots displayed the expression of CAF-associated marker genes after clustering. [file Image_1.tif]

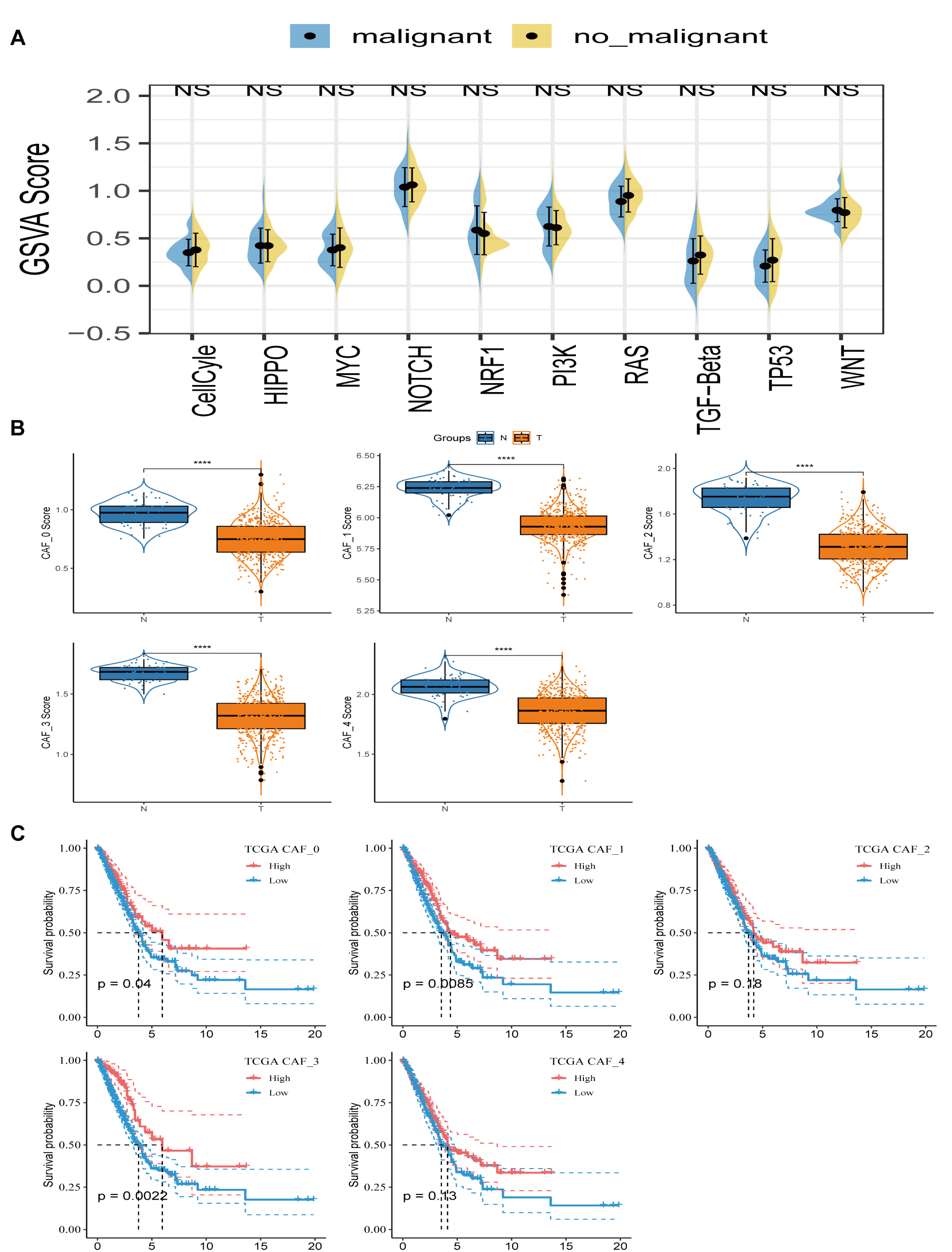

Supplement: Supplementary Figure 2 — (A) Comparison of each pathway between malignant and non-malignant cells based on GSVA score in CAF_0. (B) Comparison of five CAF scores in malignant and non-malignant tissues. (C) K-M curves of high-and-low CAF-score groups in the five clusters. [file Image_2.tif]

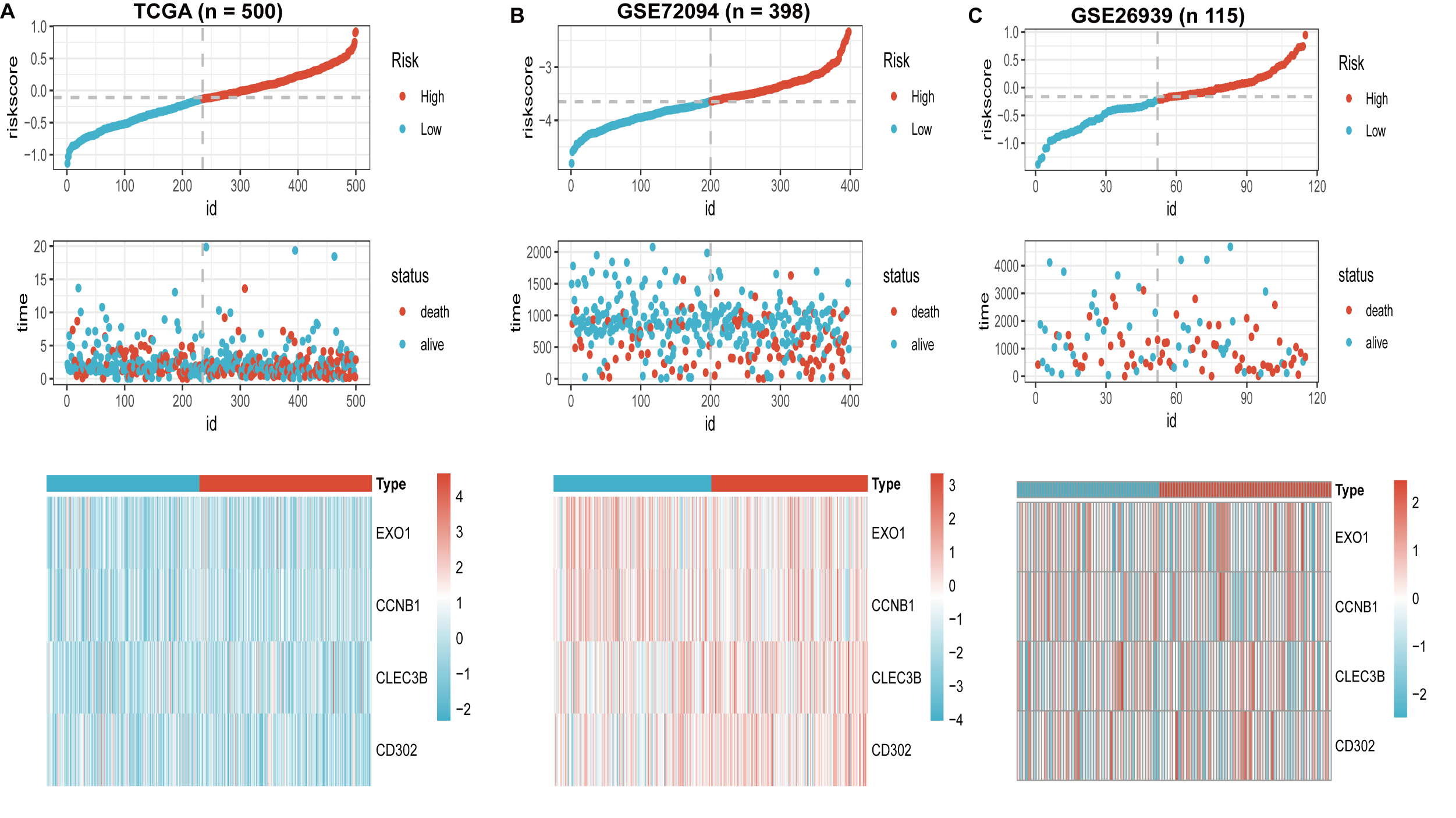

Supplement: Supplementary Figure 3 — Validation of the signature’s capacity in prognosis prediction (A) Distribution of risk scores and survival status in TCGA cohort. (B) Distribution of risk scores and survival status in GSE72094 cohort. (C) Distribution of risk scores and survival status in GSE26939. [file Image_3.tif]

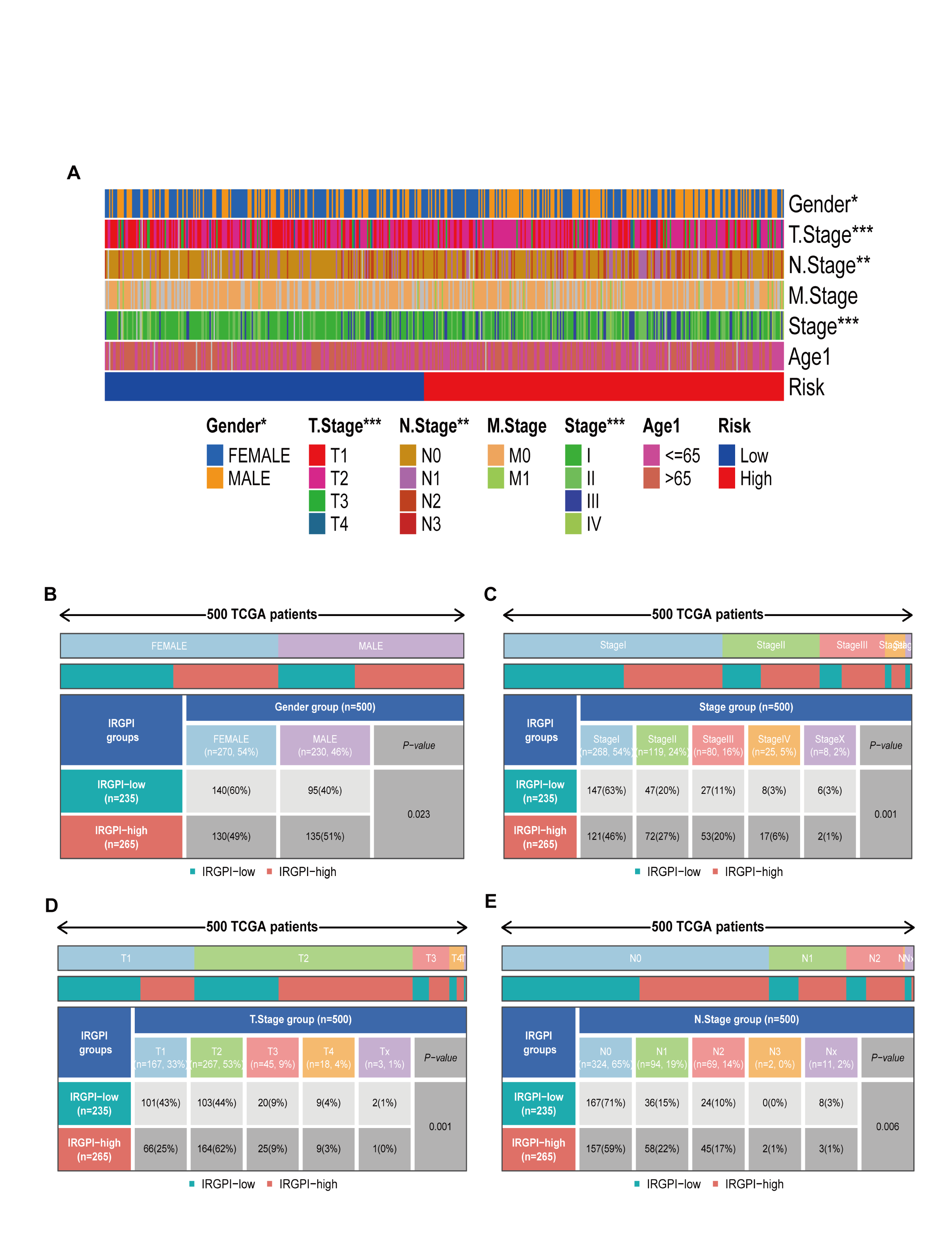

Supplement: Supplementary Figure 4 — The relationship between risk score and several clinicopathologic features. (A) The landscape of differences between high-and-low-risk subgroups. The differences between high-and-low-risk groups based on Gender (B), Stage (C), T-stage (D), N-stage (E). [file Image_4.tif]
